# Identifying pathways to religious service attendance among older adults: A lagged exposure-wide analysis

**DOI:** 10.1371/journal.pone.0278178

**Published:** 2022-11-29

**Authors:** Richard G. Cowden, Julia S. Nakamura, Zhuo Job Chen, Brendan Case, Eric S. Kim, Tyler J. VanderWeele

**Affiliations:** 1 Human Flourishing Program, Institute for Quantitative Social Science, Harvard University, Cambridge, Massachusetts, United States of America; 2 Department of Psychology, University of British Columbia, Vancouver, BC, Canada; 3 School of Nursing, University of North Carolina at Charlotte, Charlotte, NC, United States of America; 4 Lee Kum Sheung Center for Health and Happiness, Harvard T.H. Chan School of Public Health, Boston, MA, United States of America; 5 Department of Epidemiology, Harvard T.H. Chan School of Public Health, Boston, MA, United States of America; 6 Department of Biostatistics, Harvard T.H. Chan School of Public Health, Boston, MA, United States of America; Emory University, School of Public Health, UNITED STATES

## Abstract

We used prospective data (spanning 8 years) from a national sample of older U.S. adults aged > 50 years (the Health and Retirement Study, *N* = 13,771) to evaluate potential factors that lead to subsequent religious service attendance. We applied a lagged exposure-wide epidemiologic design and evaluated 60 candidate predictors of regular subsequent religious service attendance. Candidate predictors were drawn from the following domains: health behaviors, physical health, psychological well-being, psychological distress, social factors, and work. After rigorous adjustment for a rich set of potential confounders, we observed modest evidence that changes in some indices of physical health, psychological well-being, psychological distress, and social functioning predicted regular religious service attendance four years later. Our findings suggest that there may be opportunities to support more regular religious service attendance among older adults who positively self-identify with a religious/spiritual tradition (e.g., aid services for those with functional limitations, psychological interventions to increase hope), which could have downstream benefits for various dimensions of well-being in the later years of life.

## Introduction

A consistent theme across many cultures and religious traditions is that holistic well-being intersects the mind, body, and spirit [1–3]. Religion is a multidimensional concept involving beliefs, behaviors, rituals, and ceremonies related to the transcendent (e.g., God, Higher Power), and research has shown that religion may have positive or negative influences on health and well-being [4, 5]. Religious engagement often includes private (e.g., prayer, scripture reading) and public forms of participation (e.g., religious service attendance, study groups). A growing body of robust longitudinal evidence suggests that religious service attendance in particular is associated with indicators of improved physical, behavioral, and psychosocial health and well-being across adulthood, including among older adults [6–13]. For example, research suggests that more regular religious service attendance is related to lower risk of cause-specific (e.g., suicide) and all-cause mortality, improved health behaviors (e.g., reduced smoking), better psychological well-being (e.g., reduced depression), and higher social well-being (e.g., more social connectedness). Religious service attendance is also associated with virtuous and prosocial actions, such as volunteering and charitable giving [14–19], which confer benefits to broader society (e.g., reducing the social and economic burden of substance abuse and depression, providing tangible support to those in need).

Religious service attendance appears to affect well-being more strongly than other aspects of religious affiliation taken in isolation, including private prayer or doctrinal beliefs [20–22]. Although most of these findings are drawn from American samples consisting largely of Christians, similar results have been found using data on Christians in highly-secular Denmark [23], Israeli Jews [24], and a diverse mix of Taoists, Buddhists, animists, and Christians in Taiwan [22].

Despite the potential role of religious service attendance in supporting the well-being of individuals and communities, evidence indicates that religious service attendance is declining in rich, highly developed parts of the world (e.g., North America [25]). For example, estimates suggest that the percentage of U.S. adults who do not attend religious services has more than doubled over the last few decades (from 11% in the early 1970s to 26% in 2014), with the most striking declines in religious service attendance occurring after the start of the 21^st^ century [26]. This trend cannot be exclusively accounted for by population replacement, as Twenge and colleagues [26] observed a general pattern of decline in religious orientation among all generations in the U.S. population. Research suggests that this cross-generational trend may to be linked to a decrease in church membership, including a roughly 10% decline in church membership among older generations of U.S. adults (e.g., traditionalists, baby boomers) over the last two decades [27].

Many factors seem to drive religious disaffiliation and declining participation in the U.S. and elsewhere. One standard theory is that religion suffers in developed economies with high levels of education and robust social safety nets, which unsettle traditional certitudes and substitute for the social support historically provided by religious communities [28, 29]. This thesis only imperfectly accounts for trends in the U.S., however, where religious participation has declined fastest among the least educated and least affluent, which is part of a more general trend toward social disaffiliation among the lower classes (in large part due to their declining economic prospects), as evident in declining rates of marriage, unionization, and civic participation more broadly [30]. Over the past 20 years, many religious communities have also been impacted by scandal, notably over sexual abuse within churches and its subsequent cover-up by leaders, which has negatively affected public trust in religious communities and likely contributed to disaffiliation as well [31].

Given the apparent decline in religious service attendance within the U.S. (and other parts of the Western world), research is needed to identify and better understand the factors that might influence religious service attendance. Such evidence could contribute to determining suitable pathways to increase religious service attendance or identifying barriers that impede more regular religious service attendance, particularly among those who positively self-identify with a religious/spiritual tradition and subpopulations (e.g., older adults) for whom the benefits of religious services attendance might be especially pronounced. For example, advancing age is often accompanied by hardships in various domains of life, including physical (e.g., functional limitations), social (e.g., loss of loved ones), and emotional (e.g., grief) afflictions [32]. Religious service attendance can provide older adults with opportunities to access, develop, or acquire a wide range of supportive resources, both spiritual (e.g., closer connection to the divine) and nonspiritual (e.g., social relationships), which they can draw on as they encounter such adversities during their later years of life. A more thorough understanding of the potential pathways and/or barriers to religious service attendance among older adults could be a useful stepping-stone towards future research and applied work aimed at increasing religious service attendance in this subpopulation, which may precipitate additional gains in resources that support adjustment during the later years of life. In the present study, we apply a rigorous analytic template to examine an array of potential predictors of religious service attendance in a large cohort of older U.S. adults.

Prior research that has examined factors leading to an increase in religious service attendance has contributed substantially to the literature, but there are several notable gaps in the existing evidence. First, many prior studies in this area (including those with older adults) have relied on cross-sectional data, and such designs cannot infer causality [7]. For example, cross-sectional data may reveal that depression is associated with a lower likelihood of religious service attendance either because depressed people may stop attending, or because people who cease attending religious services are more likely to become depressed, which generates concerns about potential reverse causation [33]. Additionally, the observed associations in cross-sectional studies may overestimate the actual effect of an exposure on an outcome [34]. Hence, additional longitudinal evidence is needed to enrich our understanding of the strength and directionality of associations between candidate predictors and religious service attendance.

Second, existing longitudinal studies on this topic (including those involving older adults) typically address one or a narrow range of exposure variables [11, 33, 35–40]. For example, depression is one of the most frequently studied predictors of religious service attendance, but it has seldom been examined alongside other potential determinants of religious service attendance. In one of the few longitudinal studies that extended beyond psychological predictors of religious service attendance, Ferraro and Kelley-Moore [36] observed that not smoking and more frequent volunteering were associated with increased subsequent religious service attendance. However, none of the physical health indicators (e.g., chronic illness) that they examined predicted change in religious service attendance. Although these prior studies (most of which have principally applied theory-based methods to select exposures) have identified important potential determinants of religious service attendance, a more complete picture of the dynamics that shape religious service attendance among older adults may be acquired by examining a wider range of possible predictors across multiple domains of functioning. Specifically, examining an array of predictors across different domains of functioning (including those that have been considered less frequently in previous work) within the same sample may provide novel insights into the strongest predictors (as compared to many others across domains) of subsequent religious service attendance. Research along these lines could also help to build the kind of foundation needed to develop interventions that address pathways to religious service attendance in a more holistic and integrative way.

Third, longitudinal studies in this area have rarely controlled for prior values of the exposures, which can help reduce concerns about reverse causation [41]. Adjusting for prior values of the exposures can also elucidate how changes in each exposure—much like what one might expect to see from an intervention targeting that exposure—are associated with an outcome of interest. Because many potential determinants of religious service attendance (e.g., depression) may change over time, adjustment for prior values of an exposure can strengthen causal inference [42]. If such analytic decisions can be applied to improve causal inference in observational studies examining predictors of religious service attendance, researchers and practitioners are likely to be equipped with stronger evidence that they could use to make more informed decisions about how to increase religious service attendance in a particular target population and the resources they may need to allocate in order to achieve this objective.

To help enrich the existing empirical literature in this area, the purpose of the current study is to explore the determinants of change in religious service attendance among older U.S. adults. We applied a lagged exposure-wide epidemiologic design, which is a data-driven approach aimed at elucidating promising predictors of a particular outcome that can then be examined further in subsequent research. This analytic approach includes steps that rigorously control for potential confounding and dampen concerns about reverse causation [41], thereby strengthening causal inference. Using a large cohort of older U.S. adults, we test for evidence concerning potential causal effects of 60 candidate determinants of religious service attendance, including indicators of health behaviors, physical health, psychological well-being, psychological distress, social factors, and work.

## Method

### Study population

We used data from the Health and Retirement Study (HRS), a national panel study of adults aged > 50 years in the U.S. In 2006, about half of HRS respondents completed an enhanced face-to-face interview (when psychosocial data were first collected). The other half of respondents were assessed in 2008. Participants then completed a psychosocial questionnaire (response rates of 88% in 2006 and 84% in 2008), which they mailed to the University of Michigan upon completion [43]. These sub-cohorts alternate reporting on psychosocial factors, with each participant reporting psychosocial data every four years. In this study, data from the 2006 and 2008 sub-cohorts were combined to increase sample size and statistical power. Participants were excluded if they did not report psychosocial data in 2006/2008, because over half of our candidate predictors were psychosocial factors. This resulted in a final sample of 13,771 participants.

This study used data from three time points: (1) covariates were assessed in 2006/2008 (T_0_), (2) candidate predictors were assessed in 2010/2012 (T_1_), and (3) the outcome (religious service attendance) was assessed in 2014/2016 (T_2_). The HRS (see further study documentation on the HRS website: http://hrsonline.isr.umich.edu/) is sponsored by the National Institute on Aging (NIA U01AG009740) and conducted by the University of Michigan [44]. The ethics board at Harvard University exempted the present study from review because it used de-identified and publicly available data.

### Measures

#### Religious service attendance

Respondents were asked: “About how often have you attended religious services during the past year?” Response options included: > 1x/week, 1x/week, 2-3x/month, ≥ 1x/year, and never. Results for both (1) the binary religious service attendance (0 = < 1x/week, 1 = ≥ 1x/week) and (2) the ordinal religious service attendance variables (1 = rarely or never, 2 = 2-3x/month, 3 = 1x/week, 4 = > 1x/week) are available in the main text (binary variable) and supplementary materials (ordinal variable). We used the binary measure of religious service attendance (< 1x/week or ≥ 1x/week) in our main analyses because religious service attendance at the threshold of ≥ 1x/week has been associated with improved health and well-being [7]. Further, ordinal measures of religious service attendance are more difficult to interpret (as these analyses represent movement between subcategories of religious service attendance), and there was relatively little movement between categories using the ordinal variable in our study (see S1 Table, which shows the *changes* in religious service attendance from T_0_ to T_2_).

#### Covariates

We adjusted for numerous covariates assessed at T_0_, including age (continuous), gender (male, female), race/ethnicity (White, African-American, Hispanic, other), marital status (not married, married), income (< $50,000, $50,000-$74,999, $75,000-$99,999, ≥ $100,000), total wealth (based on quintiles of the score distribution for total wealth in this sample), educational attainment (no degree, GED/high school diploma, ≥ college degree), employment status (no, yes), health insurance (no, yes), geographic region (Northeast, Midwest, South, West), personality traits (openness, conscientiousness, extraversion, agreeableness, neuroticism; continuous), childhood abuse (no, yes), and religious service attendance (as either a binary or ordinal variable to match the coding of the outcome variable). To examine change in each candidate predictor, we additionally adjusted for prior values of all candidate predictors assessed at T_0_ (except for the depression variable, which was constructed from the depressive symptoms variable).

#### Predictors

We evaluated 60 candidate predictors assessed at T_1_ (2010/2012). These included measures of (1) health behaviors (frequent physical activity, smoking, heavy drinking, sleep problems); (2) physical health (diabetes, hypertension, stroke, cancer, heart disease, lung disease, arthritis, overweight/obese, physical functioning limitations, cognitive impairment, chronic pain, self-rated health, hearing, eyesight); (3) psychological well-being (positive affect, life satisfaction, optimism, purpose in life, personal mastery, health mastery, financial mastery); (4) psychological distress (depression, depressive symptoms, hopelessness, negative affect, perceived constraints, anxiety symptoms, trait anger, state anger, cynical hostility, stressful life events, financial strain, daily discrimination, major discrimination); (5) social factors (living with a spouse/partner, frequency of contact with (i) children, (ii) other family, and (iii) friends, loneliness, closeness with spouse, number of close (i) children, (ii) other family, and (iii) friends, social support from (i) spouse, (ii) children, (iii) other family, and (iv) friends, social strain from (i) spouse, (ii) children, (iii) other family, and (iv) friends, volunteering, helping friends, neighbors, or relatives, social status ladder ranking, change in social status ladder ranking); and (6) work (in the labor force). S1 Appendix and the HRS materials provide further details about each of these variables [43, 45, 46].

#### Multiple imputation

There were missing data for the covariates (up to 10.2%), candidate predictors (up to 56.2%), and outcome (33.8%). Depending on the predictor being evaluated, complete-case analyses resulted in a loss of 60.4% to 78.1% of the total sample. Thus, we imputed missing data for the covariates, candidate predictors, and outcome using imputation by chained equations, and five datasets were created. This method tends to be more flexible than other methods for handling missing data [47, 48], and it also helps address problems that arise from attrition [49, 50].

### Statistical analysis

#### Primary analysis

We used an exposure-wide analytic approach [41], with separate models estimated for each candidate predictor (see S2 Appendix). In our primary analysis, religious service attendance was a binary outcome with a prevalence ≥ 10%. Thus, we used generalized linear models (with a log link and Poisson distribution) to individually regress religious service attendance at T_2_ (2014/2016) on each candidate predictor at T_1_ (2010/2012). All continuous predictors were standardized (mean = 0, standard deviation = 1) so that their effect sizes could be interpreted as a standard deviation change in the exposure variable. We marked multiple *p*-value cut-offs in our tables (including Bonferroni-corrected), as multiple testing practices vary widely and are continuously evolving. We also report exact confidence intervals. Unless otherwise specified, we focus our discussion on the primary analytic results with religious service attendance modeled as a binary outcome in the full sample.

#### Additional analyses

We conducted several additional analyses. First, we used *E*-values to assess the minimum strength of association (on the risk ratio scale) that an unmeasured confounder would need to have with both the exposure and the outcome to explain away the observed association [51]. This helps us evaluate the robustness of the results from the primary analysis to potential unmeasured confounding. Second, we repeated the primary analysis among the subsample of participants who regularly attended religious services (≥ 1x/week) at T_1_ (S2 Table). This allowed us to identify what factors predict continued religious service attendance, four years later, for people who were already attending religious services regularly at T_1_. Third, we repeated the primary analysis among the subsample of participants who did not attend religious services regularly (< 1x/week) at T_1_ (S3 Table). This allowed us to identify what factors predict regular religious service attendance, four years later, for people who did not regularly attend religious services at T_1_. Fourth, we replicated all analyses using the abovementioned ordinal religious service attendance variable (1 = rarely or never, 2 = 2-3x/month, 3 = 1x/week, 4 = > 1x/week) by applying ordinal logistic regression, although this makes the additional assumption that the odds ratio for the covariates across each of two adjacent ordinal outcomes categories is constant. This assumption allows for greater statistical power and potential additional nuance in the results. Specifically, we repeated these analyses (1) in the entire analytic sample (S4 Table), (2) only among people who attended religious services regularly (≥ 1x/week) at T_1_ (S5 Table), and (3) only among people who did not attend religious services regularly (< 1x/week) at T_1_ (S6 Table). Fifth, we reproduced the primary analysis using complete cases to assess the impact of multiple imputation on results (S7 Table).

## Results

At T_0_, participants were on average 69 years old (*SD* = 10), a majority of whom were women (58%) and married (62%). Table 1 provides the distribution of covariates by religious service attendance at T_0_.

**Table 1:** Distribution of participant characteristics (T_0_) by religious service attendance (T_0_)^a^^,^^b^^,^^c^.

Characteristic	Religious service attendance			
	< 1x/week (*n* = 7,755)		≥ 1x/week (*n* = 6,007)	
	*n* (%)	*M* (*SD*)	*n* (%)	*M* (*SD*)
**Sociodemographic factors**				
Age (yrs; range: 52–104)		68.4 (9.8)		70.4 (9.3)
Female (%)	4175 (53.8)		3860 (64.3)	
Race/ethnicity (%)				
White	6252 (80.6)		4386 (73.0)	
Black	760 (9.8)		997 (16.6)	
Hispanic	568 (7.3)		519 (8.6)	
Other	174 (2.2)		105 (1.8)	
Married (%)	4677 (60.3)		3911 (65.1)	
Annual household income (%)				
< $50,000	4599 (59.3)		3755 (62.5)	
$50,000-$74,999	1195 (15.4)		922 (15.4)	
$75,000-$99,999	667 (8.6)		485 (8.1)	
≥ $100,000	1294 (16.7)		845 (14.1)	
Total wealth (%)				
1st Quintile	1721 (22.2)		1031 (17.2)	
2nd Quintile	1537 (19.8)		1216 (20.2)	
3rd Quintile	1489 (19.2)		1264 (21.0)	
4th Quintile	1428 (18.4)		1322 (22.0)	
5th Quintile	1580 (20.4)		1174 (19.5)	
Education (%)				
< High school	1559 (20.2)		1155 (19.3)	
High school	4186 (54.1)		3321 (55.4)	
≥ College	1990 (25.7)		1523 (25.4)	
Work				
In labor force	2882 (37.2)		1899 (31.6)	
Health insurance (%)	7382 (95.3)		5792 (96.5)	
Geographic region (%)				
Northeast	1293 (16.7)		798 (13.3)	
Midwest	1896 (24.5)		1695 (28.3)	
South	2886 (37.3)		2606 (43.4)	
West	1664 (21.5)		901 (15.0)	
Childhood abuse (%)	559 (7.3)		290 (4.9)	
**Health behaviors**				
Frequent physical activity (%)	5343 (68.9)		4521 (75.4)	
Smoking (%)	1369 (17.8)		356 (6.0)	
Heavy drinking (%)	622 (9.9)		169 (3.4)	
Sleep problems (%)	1804 (44.2)		1250 (39.4)	
**Physical health**				
Diabetes (%)	1556 (20.1)		1168 (19.5)	
Hypertension (%)	4317 (55.7)		3524 (58.7)	
Stroke (%)	670 (8.7)		439 (7.3)	
Cancer (%)	1164 (15.1)		922 (15.4)	
Heart disease (%)	1945 (25.1)		1410 (23.5)	
Lung disease (%)	886 (11.5)		416 (6.9)	
Arthritis (%)	4590 (59.3)		3708 (61.8)	
Overweight/obese (%)	5338 (69.7)		4146 (69.8)	
Physical functioning limitations (%)	2053 (26.5)		1274 (21.2)	
Cognitive impairment (%)	1501 (19.7)		1202 (20.2)	
Chronic pain (%)	2872 (37.0)		1879 (31.3)	
Self-rated health (range: 1–5)		3.1 (1.1)		3.3 (1.0)
Hearing (range: 1–5)		3.3 (1.1)		3.3 (1.1)
Eyesight (range: 1–6)		4.2 (1.0)		4.2 (1.0)
**Psychological well-being**				
Positive affect (range: 1–5)		3.5 (0.8)		3.7 (0.7)
Life satisfaction (range: 1–7)		4.9 (1.5)		5.3 (1.4)
Optimism (range: 1–6)		4.4 (1.0)		4.6 (0.9)
Purpose in life (range: 1–6)		4.5 (1.0)		4.7 (0.9)
Personal mastery (range: 1–6)		4.7 (1.1)		4.8 (1.1)
Health mastery (range: 0–10)		7.1 (2.4)		7.4 (2.3)
Financial mastery (range: 0–10)		7.2 (2.7)		7.5 (2.5)
**Psychological distress**				
Depression (%)	1228 (16.2)		651 (11.0)	
Depressive symptoms (range: 0–8)		1.6 (2.0)		1.2 (1.8)
Hopelessness (range: 1–6)		2.5 (1.3)		2.3 (1.2)
Negative affect (range: 1–5)		1.7 (0.7)		1.6 (0.6)
Perceived constraints (range: 1–6)		2.3 (1.2)		2.2 (1.2)
Anxiety symptoms (range: 1–4)		1.6 (0.6)		1.5 (0.6)
Trait anger (range: 1–4)		2.2 (0.7)		2.1 (0.7)
State anger (range: 1–4)		1.5 (0.5)		1.4 (0.5)
Cynical hostility (range: 1–6)		3.0 (1.1)		2.8 (1.1)
Stressful life events (range: 0–5)		0.2 (0.6)		0.2 (0.5)
Financial strain (range: 1–5)		2.0 (1.0)		1.9 (1.0)
Daily discrimination (range: 1–6)		1.7 (0.8)		1.6 (0.7)
Major discrimination (range: 0–6)		0.5 (0.9)		0.4 (0.9)
**Social factors**				
Living with spouse/partner (%)	4907 (65.1)		3888 (66.7)	
Contact with children (%)				
< Every few months	1199 (16.0)		644 (10.9)	
1-2x/month	926 (12.3)		584 (9.9)	
1-2x/week	2257 (30.1)		1876 (31.9)	
≥ 3x/week	3,128 (41.7)		2787 (47.3)	
Contact with other family (%)				
< Every few months	2027 (26.8)		1245 (21.3)	
1-2x/month	1788 (23.6)		1336 (22.8)	
1-2x/week	2000 (26.4)		1676 (28.7)	
≥ 3x/week	1763 (23.3)		1593 (27.2)	
Contact with friends (%)				
< Every few months	1559 (20.5)		708 (12.0)	
1-2x/month	1445 (19.0)		1035 (17.6)	
1-2x/week	2498 (32.8)		2309 (39.2)	
≥ 3x/week	2114 (27.8)		1834 (31.2)	
Loneliness (range: 1–3)		1.5 (0.6)		1.4 (0.5)
Closeness with spouse (range: 1–4)		3.4 (0.8)		3.5 (0.7)
Number of close children		2.6 (3.5)		3.0 (3.9)
Number of close other family		3.6 (5.5)		4.3 (5.7)
Number of close friends		4.0 (5.2)		5.2 (6.8)
Social support from spouse (range: 1–4)		3.4 (0.7)		3.5 (0.6)
Social support from children (range: 1–4)		3.2 (0.8)		3.4 (0.7)
Social support from other family (range: 1–4)		2.8 (0.9)		3.0 (0.8)
Social support from friends (range: 1–4)		3.0 (0.8)		3.1 (0.7)
Social strain from spouse (range: 1–4)		2.0 (0.7)		1.9 (0.7)
Social strain from children (range: 1–4)		1.7 (0.7)		1.7 (0.6)
Social strain from other family (range: 1–4)		1.6 (0.6)		1.5 (0.6)
Social strain from friends (range: 1–4)		1.8 (0.4)		1.9 (0.4)
Volunteering (%)				
0 hours	6130 (79.1)		2792 (46.5)	
1–49 hours	618 (8.0)		911 (15.2)	
50–99 hours	372 (4.8)		713 (11.9)	
100–199 hours	320 (4.1)		870 (14.5)	
≥ 200 hours	306 (4.0)		714 (11.9)	
Helping friends/neighbors/relatives (%)				
0 hours	3976 (51.4)		2644 (44.2)	
1–49 hours	1792 (23.2)		1425 (23.8)	
50–99 hours	919 (11.9)		916 (15.3)	
100–199 hours	602 (7.8)		588 (9.8)	
≥ 200 hours	450 (5.8)		409 (6.8)	
Social status ladder (range: 1–10)	6.5 (1.8)		6.6 (1.7)	
Change in social status ladder (%)				
Moved down	794 (10.6)		474 (8.3)	
No change	5742 (76.7)		4549 (79.2)	
Moved up	947 (12.7)		721 (12.6)	
**Personality traits**				
Openness (range: 1–4)		3.0 (0.6)		2.9 (0.5)
Conscientiousness (range: 1–4)		3.3 (0.5)		3.4 (0.5)
Extraversion (range: 1–4)		3.1 (0.6)		3.3 (0.5)
Agreeableness (range: 1–4)		3.5 (0.5)		3.6 (0.4)
Neuroticism (range: 1–4)		2.1 (0.6)		2.0 (0.6)

*Note*. *M* = mean, *SD* = standard deviation.

^a^Table based on non-imputed data (*N* = 13,762).

^b^All variables were assessed in 2006/2008 (T_0_) and used as covariates.

^c^Cumulative percentage for some variables may not add up to 100% due to rounding.

### Primary analysis

Results for the exposure-wide analysis involving candidate predictors of regular subsequent religious service attendance are reported in Table 2. There was little evidence of associations between health behaviors and regular subsequent religious service attendance.

**Table 2:** Candidate predictors (T_1_) of subsequent religious service attendance (T_2_)^a^^,^^b^^,^^c^.

Candidate predictor	Risk ratio	95% confidence interval
**Health behaviors**		
Frequent physical activity	1.06	0.99, 1.14
Smoking	0.80	0.58, 1.09
Heavy drinking	0.94	0.79, 1.13
Sleep problems	1.00	0.94, 1.07
**Physical health**		
Diabetes	0.99	0.87, 1.12
Hypertension	1.00	0.87, 1.15
Stroke	1.06	0.92, 1.22
Cancer	1.03	0.90, 1.17
Heart disease	0.98	0.86, 1.11
Lung disease	0.86	0.71, 1.05
Arthritis	0.97	0.85, 1.09
Overweight/obese	1.05	0.94, 1.17
Physical functioning limitations	0.84	0.77, 0.92***
Cognitive impairment	0.93	0.86, 1.01
Chronic pain	0.98	0.92, 1.05
Self-rated health	1.09	1.05, 1.13***
Hearing	1.04	1.00, 1.08
Eyesight	1.01	0.98, 1.05
**Psychological well-being**		
Positive affect	1.06	1.02, 1.11**
Life satisfaction	1.05	1.02, 1.09**
Optimism	1.04	1.00, 1.09
Purpose in life	1.06	1.00, 1.11*
Personal mastery	1.02	0.99, 1.06
Health mastery	1.05	1.01, 1.09*
Financial mastery	1.03	0.99, 1.07
**Psychological distress**		
Depression	0.94	0.84, 1.04
Depressive symptoms	0.96	0.92, 1.01
Hopelessness	0.94	0.90, 0.98**
Negative affect	0.97	0.92, 1.02
Perceived constraints	0.94	0.90, 0.98**
Anxiety symptoms	0.97	0.92, 1.01
Trait anger	0.97	0.94, 1.01
State anger	0.96	0.92, 1.00
Cynical hostility	1.00	0.96, 1.03
Stressful life events	0.98	0.95, 1.01
Financial strain	0.99	0.96, 1.03
Daily discrimination	1.00	0.96, 1.04
Major discrimination	1.00	0.96, 1.05
**Social factors**		
Living with spouse/partner	1.03	0.92, 1.14
Contact with children		
< Every few months	Reference	Reference
1-2x/month	1.03	0.89, 1.20
1-2x/week	1.01	0.88, 1.15
≥ 3x/week	0.97	0.85, 1.11
Contact with other family		
< Every few months	Reference	Reference
1-2x/month	0.98	0.88, 1.09
1-2x/week	0.96	0.86, 1.08
≥ 3x/week	0.98	0.86, 1.13
Contact with friends		
< Every few months	Reference	Reference
1-2x/month	1.13	1.02, 1.26*
1-2x/week	1.19	1.08, 1.31***
≥ 3x/week	1.19	1.06, 1.33**
Loneliness	1.00	0.95, 1.04
Closeness with spouse	1.02	0.95, 1.10
Number of close children	1.01	0.98, 1.04
Number of close other family	1.01	0.98, 1.04
Number of close friends	1.01	0.98, 1.04
Social support from spouse	1.00	0.95, 1.04
Social support from children	1.00	0.96, 1.05
Social support from other family	1.01	0.97, 1.06
Social support from friends	1.04	1.00, 1.08*
Social strain from spouse	1.01	0.97, 1.06
Social strain from children	1.00	0.96, 1.03
Social strain from other family	1.00	0.95, 1.05
Social strain from friends	1.04	1.00, 1.07*
Volunteering		
0 hours	Reference	Reference
1–49 hours	1.32	1.20, 1.45***
50–99 hours	1.35	1.22, 1.49***
100–199 hours	1.40	1.26, 1.55***
≥ 200 hours	1.39	1.23, 1.57***
Helping friends/neighbors/relatives		
0 hours	Reference	Reference
1–49 hours	1.07	0.99, 1.16
50–99 hours	1.07	0.97, 1.18
100–199 hours	1.07	0.95, 1.21
≥ 200 hours	1.07	0.93, 1.22
Social status ladder	1.01	0.97, 1.05
Change in social status ladder		
Moved down	Reference	Reference
No change	1.02	0.92, 1.12
Moved up	1.01	0.89, 1.15
**Work**		
In labor force	0.95	0.87, 1.05

*Note*. *N* = 13,771 for all analyses.

*p < .05 before Bonferroni correction

** *p* < .01 before Bonferroni correction

***p < .05 after Bonferroni correction (the *p*-value cut-off for Bonferroni correction is *p* = .05/60 predictors: *p* < .00083333).

^a^The analytic sample was restricted to those who had participated in 2006/2008 (T_0_). Multiple imputation was performed to impute missing data on the covariates, candidate predictors, and outcome. Candidate predictors were assessed in 2010/2012 (T_1_), and the outcome (religious service attendance) was assessed in 2014/2016 (T_2_). All models controlled for the full set of covariates, including sociodemographic factors, personality traits, prior values of all candidate predictors (except for the depression variable, which was constructed from the depressive symptoms variable), and prior religious service attendance, each of which was assessed at T_0_ (see Table 1).

^b^All continuous candidate predictors were standardized (mean = 0, standard deviation = 1).

^c^An exposure-wide analytic approach was used, and a separate model was run for each candidate predictor. Because religious service attendance was a binary outcome with a prevalence of ≥ 10%, we ran a generalized linear model with a log link and Poisson distribution to estimate a risk ratio.

For physical health factors, having physical functioning limitations was associated with a 16% lower likelihood of attending religious services regularly (95% CI: 0.77, 0.92) four years later. For each standard deviation increase in self-rated health, participants were 9% more likely to subsequently attend religious services regularly (95% CI: 1.05, 1.13). However, there was little evidence of associations between other physical health indicators and regular subsequent religious service attendance.

Among psychological well-being factors, participants were more likely to regularly attend religious services four years later for each standard deviation increase in positive affect (RR = 1.06; 95% CI: 1.02, 1.11), life satisfaction (RR = 1.05; 95% CI: 1.02, 1.09), purpose in life (RR = 1.06; 95% CI: 1.00, 1.11), and health mastery (RR = 1.05; 95% CI: 1.01, 1.09). Among psychological distress factors, participants were 6% less likely to regularly attend religious services four years later for each standard deviation increase in hopelessness (95% CI: 0.90, 0.98) and perceived constraints (95% CI: 0.90, 0.98). There was little evidence of associations between other psychological well-being and distress factors and regular subsequent religious service attendance.

For social factors, participants who were in contact with friends 1-2x/month (RR = 1.13; 95% CI: 1.02, 1.26), 1-2x/week (RR = 1.19; 95% CI: 1.08, 1.31), or ≥ 3x/week (RR = 1.19; 95% CI: 1.06, 1.33) were more likely to regularly attend religious services four years later. For each standard deviation change in positive social support from friends (95% CI: 1.00, 1.08) and social strain from friends (95% CI: 1.00, 1.07), participants were 4% more likely to subsequently attend religious services regularly. Participants who volunteered 1–49 hours/year (RR = 1.32; 95% CI: 1.20, 1.45), 50–99 hours/year (RR = 1.35; 95% CI: 1.22, 1.49), 100–199 hours/year (RR = 1.40; 95% CI: 1.26, 1.55), and ≥ 200 hours/year (RR = 1.39; 95% CI: 1.23, 1.57) were more likely to regularly attend religious services four years later. There was little evidence of associations between other social factors and regular subsequent religious service attendance. Work was also not associated with regular subsequent religious service attendance.

### Additional analyses

*E*-values suggested that many of the observed associations were moderately robust to unmeasured confounding (Table 3). To illustrate, for physical functioning limitations, an unmeasured confounder associated with both physical functioning limitations and religious service attendance by risk ratios of 1.66 each (above and beyond the covariates already adjusted for) could explain away the association, but weaker joint confounding associations could not. Further, to shift the confidence interval to include the null, an unmeasured confounder associated with both physical functioning limitations and religious service attendance by risk ratios of 1.39 could suffice, but weaker joint confounding associations could not.

**Table 3:** Robustness to unmeasured confounding (*E*-values) for the associations between candidate predictors (T_1_) and subsequent religious service attendance (T_2_)^a^^,^^b^^,^^c^.

Candidate predictor	Effect estimate^b^	Confidence interval limit^c^
**Health behaviors**		
Frequent physical activity	1.32	1.00
Smoking	1.82	1.00
Heavy drinking	1.31	1.00
Sleep problems	1.05	1.00
**Physical health**		
Diabetes	1.10	1.00
Hypertension	1.05	1.00
Stroke	1.32	1.00
Cancer	1.19	1.00
Heart disease	1.17	1.00
Lung disease	1.59	1.00
Arthritis	1.23	1.00
Overweight/obese	1.29	1.00
Physical functioning limitations	1.66	1.39
Cognitive impairment	1.36	1.00
Chronic pain	1.15	1.00
Self-rated health	1.39	1.26
Hearing	1.23	1.00
Eyesight	1.11	1.00
**Psychological well-being**		
Positive affect	1.33	1.16
Life satisfaction	1.29	1.15
Optimism	1.26	1.00
Purpose in life	1.30	1.02
Personal mastery	1.17	1.00
Health mastery	1.28	1.12
Financial mastery	1.21	1.00
**Psychological distress**		
Depression	1.34	1.00
Depressive symptoms	1.24	1.00
Hopelessness	1.32	1.15
Negative affect	1.22	1.00
Perceived constraints	1.32	1.16
Anxiety symptoms	1.23	1.00
Trait anger	1.20	1.00
State anger	1.25	1.00
Cynical hostility	1.05	1.00
Stressful life events	1.18	1.00
Financial strain	1.10	1.00
Daily discrimination	1.05	1.00
Major discrimination	1.06	1.00
**Social factors**		
Living with spouse/partner	1.19	1.00
Contact with children		
< Every few months	Reference	Reference
1-2x/month	1.22	1.00
1-2x/week	1.09	1.00
≥ 3x/week	1.20	1.00
Contact with other family		
< Every few months	Reference	Reference
1-2x/month	1.18	1.00
1-2x/week	1.23	1.00
≥ 3x/week	1.14	1.00
Contact with friends		
< Every few months	Reference	Reference
1-2x/month	1.53	1.17
1-2x/week	1.65	1.36
≥ 3x/week	1.66	1.30
Loneliness	1.05	1.00
Closeness with spouse	1.16	1.00
Number of close children	1.12	1.00
Number of close other family	1.10	1.00
Number of close friends	1.11	1.00
Social support from spouse	1.05	1.00
Social support from children	1.05	1.00
Social support from other family	1.12	1.00
Social support from friends	1.24	1.05
Social strain from spouse	1.13	1.00
Social strain from children	1.07	1.00
Social strain from other family	1.02	1.00
Social strain from friends	1.24	1.06
Volunteering		
0 hours	Reference	Reference
1–49 hours	1.96	1.69
50–99 hours	2.04	1.74
100–199 hours	2.14	1.82
≥ 200 hours	2.13	1.76
Helping friends/neighbors/relatives		
0 hours	Reference	Reference
1–49 hours	1.36	1.00
50–99 hours	1.34	1.00
100–199 hours	1.36	1.00
≥ 200 hours	1.33	1.00
Social status ladder	1.12	1.00
Change in social status ladder		
Moved down	Reference	Reference
No change	1.15	1.00
Moved up	1.11	1.00
**Work**		
In labor force	1.27	1.00

*Note*. *N* = 13,771 for all analyses.

^a^See VanderWeele and Ding (2017) for the formula for calculating *E*-values.

^b^The *E*-values for effect estimates are the minimum strength of association on the risk ratio scale that an unmeasured confounder would need to have with both the exposure and the outcome to fully explain away the observed association between the exposure and outcome, conditional on the measured covariates.

^c^The *E*-values for the limit of the 95% confidence interval closest to the null denote the minimum strength of association on the risk ratio scale that an unmeasured confounder would need to have with both the exposure and the outcome to shift the confidence interval to include the null value, conditional on the measured covariates.

Results were somewhat different (see S2 and S3 Tables) when comparing candidate predictors for the subsample of participants who attended religious services regularly at T_1_ vs. those who did not. For example, some indices of physical health (e.g., cancer), psychological well-being (e.g., positive affect), and psychological distress (e.g., hopelessness) were predictive of regular subsequent attendance among participants who did not attend regularly at T_1_, but not among those who did attend regularly at T_1_. When religious service attendance was modeled as an ordinal variable, results for the entire sample, among participants who attended religious services regularly at T_1_, and among those who did not attend religious services regularly at T_1_ showed some differences from each other as well as from results of analyses in which religious service attendance was modeled as a binary variable (see S4–S6 Tables for the entire analytic sample, for those who did regularly attend religious services at T_1_, and for those who did not regularly attend religious services at T_1_, respectively). For example, when using the ordinal religious service attendance variable with the full analytic sample (see S4 Table), we observed associations for some predictors (e.g., frequent physical activity, cognitive impairment, optimism, state anger, helping friends, neighbors, or relatives) that were not associated with subsequent religious service in the primary analysis. This is not entirely surprising, given that ordinal logistic regression tends to increase power within analyses (though at the cost of additional assumptions). The odds ratios in this analysis concern the odds ratio for each T_1_ predictor comparing two adjacent religious service attendance categories at T_2_ (e.g., rarely or never compared to 2-3x/month, or 2-3x/month compared to 1x/week, or 1x/week compared to > 1x/week). For odds ratios comparing non-adjacent categories, one exponentiates the odds ratio in the table by how many steps the categories are apart. Results of the complete-case analyses were somewhat similar to the results we observed in the primary analysis (see S7 Table).

## Discussion

Using longitudinal data from a prospective sample of older U.S. adults, we evaluated potential determinants of regular (≥ 1x/week) religious service attendance across several domains, including health behavior, physical health, psychological well-being, psychological distress, social factors, and work. In the primary analysis, we observed that *changes* in some physical health (i.e., physical functioning limitations, self-rated health), psychological well-being (i.e., positive affect, life satisfaction, purpose in life, health mastery), psychological distress (i.e., hopelessness, perceived constraints), and social factors (i.e., contact with friends, positive social support from friends, negative social strain from friends, volunteering) were associated with regular subsequent service attendance four years later. Changes in several predictors that have seldom received attention in this literature (e.g., health mastery) were associated with subsequent religious service attendance in our sample of older adults.

### Health behaviors and physical health

Our results did not provide substantial evidence of associations between health behaviors (e.g., frequent physical activity, heavy drinking) and regular subsequent religious service attendance. These findings diverge from some prior longitudinal research. For example, status as a smoker has been modestly associated with a lower likelihood of subsequent religious service attendance among younger and older adults [36]. Although the effect estimate we observed for smoking in the full analytic sample was similar to effect estimates reported in previous work, the confidence interval was wide. This is consistent with the possibility of a null or modest effect of smoking on regular service attendance in the older U.S. adult population, which is because levels of smoking were relatively stable over time in this sample. Additional research involving a wide range of health behaviors in younger samples and over longer periods of time could clarify whether changes in health behaviors might lead to more frequent religious service attendance.

Study participants who reported improvements in physical functioning limitations and self-rated health were more likely to regularly attend religious services four years later. Compared to some earlier work involving older US adults [52], our findings suggest that functional disabilities might have longer-term effects on religious service attendance than previously found. However, none of the other indices of physical health status that we evaluated (e.g., hearing, eyesight) were associated with regular subsequent religious service attendance. Taken together, this study’s fundings indicate that difficulties performing more basic physical activities (e.g., getting up from a chair, walking across a room) and a general decline in physical health that typically comes with aging may make it challenging for some older adults to regularly attend religious services in the longer-term. In light of the potential benefits that older adults might glean from “the fellowship and inspirational experiences” (p. 246) that accompany religious service attendance [53], professional care services (e.g., mobility support) and onsite assistance by communities of faith could enable older adults with functional limitations to attend religious services more regularly.

We found little evidence of association between the chronic conditions examined in this study (e.g., diabetes, hypertension) and regular subsequent religious service attendance. These findings resonate with previous longitudinal research [36] that showed no association of religious service attendance with either generalized chronic illness categories (e.g., circulatory conditions) or specific chronic illnesses (e.g., cancer diagnosis). However, one interesting exception emerged from the stratified analyses; cancer diagnosis was associated with a higher likelihood of regular subsequent religious service attendance among participants who did not attend regularly at T_1_ (see S3 Table). This finding may reflect the *stress mobilization effect* [54, 55], which refers to the notion that negative life events can trigger religious/spiritual coping responses (e.g., seeking comfort from God by attending religious services). An alternative but complementary explanation is that irregular attenders at T_1_ who lived with and survived cancer over the four-year study period may have gone through a process of religious/spiritual transformation that is reflected in more frequent faith-based activities, such as regular religious service attendance [56]. Interestingly, no other chronic illnesses that we assessed were associated with subsequent religious service attendance among those who did not attend regularly at T_1_. This may be because cancer is one of the most feared chronic illnesses among older adults [57], and it is possible that the perceived existential threat of cancer precipitates stronger change in religious/spiritual involvement when compared to other kinds of serious illness. In future research, subgroup analyses stratified by prior attendance levels may be of practical interest in identifying those who might benefit from initiatives to support their spiritual/religious response to cancer and other life-threatening health conditions.

### Psychological well-being and psychological distress

Among the indices of psychological well-being that were examined in this study, we found that increases in some aspects of hedonic well-being (i.e., positive affect, life satisfaction) were associated with a higher likelihood of regular religious service attendance at follow-up. These findings are consistent with the idea that positive subjective experiences tend to prompt approach behavior (e.g., exploration of novel situations, participation in activities [58], which might lead some older adults to increase their engagement with the environment by attending religious services on a regular basis. There was also evidence that increases in some aspects of eudaimonic well-being (i.e., purpose in life, health mastery) were associated with regular subsequent religious service attendance. One possible explanation for these findings is that eudaimonic experiences, such as a renewed or enhanced sense of purpose in life, often motivate people to make positive lifestyle choices aimed at nurturing their well-being [59], including an increase in religious participation in view of the psychosocial and spiritual goods it often affords [60, 61]. Furthermore, older adults often shift their focus from the ‘resumé virtues’ that helped them succeed professionally to the ‘eulogy virtues’ by which they hope to be remembered [62]; it would be no surprise if this shift in priorities prompted a heightened interest in one’s spiritual life as well.

Of the indices of psychological distress that were examined, increases in hopelessness and perceived constraints about personal control were associated with a lower likelihood of regular subsequent religious service attendance. These findings coincide with evidence from previous cross-sectional research [63, 64], but it is not possible to discern the direction of causation from such cross-sectional data. Given the afflictions that tend to befall people in the later stages of life (e.g., deterioration of health, loss of spouses and/or friends), our findings resonate with recent calls for public health activities to aim at bolstering the psychological well-being of older adults [65]. For example, several evidenced-based programs oriented towards promoting hope have received empirical support for use in older populations [66], some of which could be adapted for large-scale implementation. Beyond the potential psychological benefits that might accompany such interventions, the findings of this study suggest that improvements in certain aspects of psychological well-being (e.g., hope, internal locus of control) may lead to an increase in regular service attendance. In turn, robust longitudinal evidence has shown that religious service attendance could have benefits for health and well-being in a variety of life domains [7–9, 11, 12].

None of the other indices of psychological distress were associated with regular subsequent religious service attendance, including depression. This latter finding warrants specific consideration because there has been substantial scholarly interest in examining the effect of depression on religious service attendance, with prior longitudinal research demonstrating mixed results [33, 35]. There could be many methodological reasons underlying the divergent results seen across studies, such as sample composition (e.g., previous research suggests that depression is more strongly associated with religious service attendance at younger ages [37]) and measurement of depression (e.g., diagnostic interview versus self-report assessment). An important contribution of this study is that we estimated the associations of both depression and hopelessness with religious service attendance, and we also simultaneously adjusted for both variables in all models, neither of which has been done in previous longitudinal research. Drawing on theory and some empirical evidence that suggests hopelessness can be a sufficient proximal cause of a subtype of depression that is referred to as *hopelessness depression* [67, 68], future research should ensure that assessment of depression is not conflated with hopelessness (or lack of hope). For example, the Center for Epidemiologic Studies Depression Scale 10, which has been used in prior research on religious service attendance and depression [11], has an item that captures a lack of hope about the future. If hopelessness (or a lack of hope) is integrated into measures of depression, it is difficult to pinpoint whether depression or hopelessness (or both) contribute to reduced religious service attendance.

### Social factors and work

Compared to all other candidate determinants examined in this study, we found that hours spent volunteering was the strongest predictor of regular subsequent religious service attendance in the full analytic sample. Our results correspond with prior longitudinal research that reported a positive association between more frequent voluntary association participation and religious service attendance [18, 36]. Collectively, these findings suggest that interventions and public health campaigns designed to encourage volunteering among older adults could lead to an increase in religious service attendance. Given the potential for volunteering to support healthy aging in multiple areas of life (e.g., physical health, psychological well-being [69], there may be other important benefits that arise from resources that are dedicated toward encouraging volunteering among older adults (e.g., economic value of volunteer activities [70]). The results of this study may also be of interest to communities of faith, as community service or outreach projects that they organize to benefit society at large could also provide them with opportunities to reach new members and further contribute to the spiritual lives of older adults.

Complementing previous longitudinal studies that have focused on aspects of social well-being as outcomes of religious service attendance among older adults (e.g., social support, social integration [7, 71], we found that an increase in both social contact with friends and social support from friends were associated with a higher likelihood of regular subsequent religious service attendance. These findings suggest that friendships during the later years of life could play an important role in whether older adults attend religious services on a regular basis. For example, older adults who are unable to drive often rely on their friends for transportation to and from religious services and other activities that take place within religious communities [72]. Interestingly, there was also evidence indicating that an increase in social strain from friends was associated with regular subsequent religious service attendance. This finding may reflect religious coping, with older adults who experience strained friendships possibly turning to the religious realm as a source of comfort for dealing with relational difficulties [73] or as an alternative resource because the quality of resources in the social domain have diminished [74]. In alignment with some prior longitudinal research involving middle-aged and older adult participants [36], we found little evidence of association between work and subsequent religious service attendance. With some research indicating that entry into the labor force predicts a decline in religious service attendance among younger adult samples [75], religious service attendance could be more strongly affected by employment changes that accompany transitional periods at younger ages (e.g., transition from adolescence to adulthood). Further research is needed to better understand the impact of life course transitions on patterns of religious service attendance within adulthood.

### Limitations, strengths, and future research

This study had several limitations. First, the sample included U.S. adults aged 50 years and older, a population for which some determinants of religious service attendance documented in prior research (e.g., living with a school-age child [39]) are largely irrelevant. Hence, it would be worthwhile to replicate this exposure-wide analysis in younger cohorts. It could also be interesting to extend this kind of analysis to other parts of the world (e.g., Africa) where religions such as Christianity and Islam are experiencing rapid growth within comparatively younger populations [76], and to differentiate between those who are religiously affiliated and those who are not. Second, this study addressed a single dimension of religiosity (i.e., religious service attendance) that does not provide a complete account of religious/spiritual life and is more relevant to the practices of some religious traditions than others. Our understanding of what determines religious involvement, including whether specific factors are common predictors of different dimensions of religiosity, would be enhanced by methodologically rigorous research focused on other aspects of religious/spiritual life. Third, four years of follow-up data may not be sufficient to detect effects for antecedents of religious service attendance that vary or accumulate over time. For example, there was little evidence of an association between loneliness and subsequent religious service attendance. If loneliness has a more pronounced effect on religious service attendance earlier in life, the data and analytic approach used in this study would not be able to detect this effect. Fourth, the findings of this study are limited by our use of self-report data. Although many potential determinants were assessed across several domains of human life, future research could build on this study by integrating objective psychological testing and markers of physical health into measurement procedures. Fifth, a concern with using observational data is that estimated effects might be biased by unmeasured confounding (e.g., intrinsic religiosity) or reverse causality. Our analytic design choices attempted to reduce concerns about unmeasured confounding and reverse causality, and *E*-values for the estimated effects indicated that the observed associations (e.g., volunteering) were at least somewhat robust to unmeasured confounding. However, we cannot rule out the possibility that the observed effects could be explained away by some combination of statistical uncertainty and unmeasured confounding.

Despite selected limitations, this study used a robust analytic approach to adduce evidence of potential causal effects of numerous candidate predictors of subsequent religious service attendance. Other strengths of our study include our ability to evaluate many understudied predictors of religious service attendance and use of a large, national sample of older adults. Although the results that emerged for selected aspects of physical health, psychological well-being, psychological distress, and social factors are certainly of interest, of equal interest is that there was little evidence that other factors, of those examined, were associated with regular subsequent religious service attendance. It may be that religious service attendance is less malleable or changeable later in life than earlier in life, and further research is needed to investigate this possibility.

## Conclusion

This prospective cohort study of older U.S. adults provided modest evidence indicating that improvements in some aspects of functioning in different domains of life (i.e., physical psychological, social) were associated with regular service attendance four years later. Our results suggest that there may be opportunities to support more regular religious service attendance among older adults who positively self-identify with a religious/spiritual tradition (e.g., aid services for those with functional limitations, psychological interventions to increase hope), which itself may confer additional benefits for various dimensions of well-being in the later years of life. Notwithstanding the findings of this study, further research is needed to identify and understand the factors that might influence changes in religious service attendance over time and throughout the life course.

## Supporting information

S1 Appendix Assessment of candidate predictors. (DOCX)

S2 Appendix Proof illustrating how adjusting for the prior value of a predictor can help us evaluate how “changes” in the predictor are associated with subsequent religious service attendance. (DOCX)

S1 Table Changes in religious service attendance from T_0_ to T_2_. (DOCX)

S2 Table Candidate predictors (T_1_) of subsequent religious service attendance (T_2_) among those who regularly attended religious services at T_1_. (DOCX)

S3 Table Candidate predictors (T_1_) of subsequent religious service attendance (T_2_) among those who did not regularly attend religious services at T_1_. (DOCX)

S4 Table Candidate predictors (T_1_) of subsequent religious service attendance (T_2_) with religious service attendance as an ordinal variable. (DOCX)

S5 Table Candidate predictors (T_1_) of subsequent religious service attendance (T_2_) among those who regularly attended religious services at T_1_ (religious service attendance modeled as an ordinal variable). (DOCX)

S6 Table Candidate predictors (T_1_) of subsequent religious service attendance (T_2_) among those who did not regularly attend religious services at T_1_ (religious service attendance modeled as an ordinal variable). (DOCX)

S7 Table Complete-case analyses for candidate predictors (T_1_) of subsequent religious service attendance (T_2_). (DOCX)
