# Supplementary material for: Identifying pathways to religious service attendance among older adults: A lagged exposure-wide analysis
Source: PLoS One. 2022 Nov 29;17(11):e0278178. doi: 10.1371/journal.pone.0278178 (PMC9707744; doi:10.1371/journal.pone.0278178)
Supplement: S1 Appendix — (DOCX) [file pone.0278178.s001.docx]

Identifying Pathways to Religious Service Attendance: A Lagged Exposure-Wide Analysis in a Sample of Older U.S. Adults

**S1 APPENDIX**

**Assessment of Candidate Predictors**

**Reference Group**

Unless otherwise noted, the reference group was the healthiest group for all binary predictors.

**Physical Health­­­­**

*Chronic conditions.* Participants self-reported (yes/no) if they were ever told by a healthcare provider that they had the following conditions: 1) diabetes, 2) hypertension, 3) stroke, 4) cancer, 5) heart disease, 6) lung disease, or 7) arthritis. The Health and Retirement Study (HRS) has demonstrated validity and reliability of self-reported chronic conditions (Fisher et al., 2005).

*Overweight/obese*. Body mass index (BMI) was derived from self-reported height and weight. It was calculated as weight/height^2^ (kg/m^2^). A BMI of ≥ 25 kg/m^2^ was considered as overweight/obese (World Health Organization, 1995).

*Physical functioning limitations.* Physical functioning limitations were assessed using items from several scales (Katz et al., 1963; Lawton & Brody, 1969; Nagi, 1976; Rosow & Breslau, 1966). A total of 15 questions about physical functioning (e.g., walking several blocks, climbing one flight of stairs, pushing or pulling large objects, lifting or carrying 10 pounds, getting up from a chair, reaching or extending arms up, stooping, kneeling, or crouching, sitting for 2 hours) and activities of daily living (e.g., walking across a room, dressing, eating, bathing, getting in or out of bed, using the toilet, picking up a dime) were included. Participants were classified as having “physical functioning limitations” if they reported > 4 limitations with physical functioning, while participants who reported ≤ 4 limitations were considered “normal” (the reference group). This criterion was determined by identifying the physical function score where 75% of participants could be considered as having healthy physical function in the HRS sample.

*Cognitive impairment*. The HRS cognitive functioning assessment (Fisher et al., 2017; Ofstedal et al., 2005) was adapted from the modified Telephone Interview for Cognitive Status. The assessment included an immediate and delayed 10-noun free recall test, a serial 7 subtraction test, and a backward count 20 test (27-point scale overall). This assessment tool has been shown to have high sensitivity and specificity when assessing cognitive impairment in older adults. The cut-off points used in this study were derived from previous research on cognitive impairment in HRS (Crimmins et al., 2011; Langa et al., 2005). Participants who scored 0-11 (on the 27-point scale) were classified as having “cognitive impairment,” while participants who scored ≥ 12 were classified as “normal” (the reference group). HRS reports contain further information about these cognitive assessments (Fisher et al., 2017; Ofstedal et al., 2005).

*Chronic pain*. Participants were asked (yes/no), “Are you often troubled with pain?” The reference group was “no” pain.

*Self-rated health.* Participants responded to the item, “Would you say your health is excellent, very good, good, fair, or poor?” using a 5-point scale (reverse coded with higher scores indicating higher self-rated health).

*Hearing.* Participants responded to the item, “Is your hearing excellent, very good, good, fair, or poor (using a hearing aid as usual)?” using a 5-point response scale (reverse coded with higher values indicating better hearing). Self-report measures of hearing have been found to be reliable measures of hearing impairment (Chou et al., 2011).

*Eyesight.* Participants were asked, “Is your eyesight excellent, very good, good, fair, or poor using glasses or corrective lenses as usual?” Response categories were as follows: 1) Excellent, 2) Very good, 3) Good, 4) Fair, 5) Poor or 6) Legally blind. Responses were reverse coded such that higher values were associated with better eyesight.

**Health Behaviors**

*Frequent physical activity*. Based on prior research, a binary physical activity variable was created: ≥ 1x/week of vigorous or moderate exercise was considered frequent physical activity, while < 1x/week of vigorous or moderate exercise was the reference group (Nandi et al., 2014). Participants indicated the frequency (i.e., response categories: daily, > 1x/week, 1x/week, 1-3x/month, hardly ever or never) with which they engaged in vigorous (e.g., running, swimming, aerobics), moderate (e.g., gardening, dancing, walking at a moderate pace), and light (e.g., vacuuming, laundry) activities over the past 12 months.

*Smoking*. Participants were asked (yes/no), “Do you smoke cigarettes now?” to assess current smoking status. The reference group was “no” smoking.

*Heavy drinking.* Following the National Institute on Alcohol Abuse and Alcoholism guidelines, heavy drinking was defined as > 14 drinks/week for men and > 7 drinks/week for women (National Institute on Alcohol Abuse and Alcoholism, n.d.). Alcohol consumption was measured by multiplying the number of days/week that alcohol was consumed x number of drinks/day, which resulted in the number of drinks/week. Participants not in this alcohol consumption range were classified as non-heavy drinkers (the reference group).

*Sleep problems.* Participants completed the 4-item Jenkins Sleep Questionnaire, a widely used and validated screening instrument for assessing sleep complaints and insomnia symptoms (Jenkins et al., 1988). Response categories included “most of the time,” “sometimes,” and “rarely or never.” Having sleep problems was defined as reporting: “most of the time” for any of the three negatively worded items (e.g., “How often do you have trouble falling asleep?”) and “rarely or never” to the one positively worded item (i.e., “feel really rested when you wake up in the morning”). Participants were considered unhealthy (i.e., having sleep problems) if they reported one or more sleep problems. The sleep questionnaire was only administered every other wave. Thus, sleep data was imputed for half of the sample.

**Psychological Well-Being**

*Positive affect.* Positive affect was measured (in 2006 only) with a 6-item scale (Mroczek & Kolarz, 1998; Watson et al., 1988) originally developed for use in the Midlife in the United States Study. The scale assessed how often the participant felt “cheerful,” “in good spirits,” “extremely happy,” “calm and peaceful,” “satisfied,” and “full of life” over the past 30 days. Response categories ranged from 1 (all of the time) to 5 (none of the time). Responses were reverse scored, so that a higher score indicated higher positive affect. An overall score was derived by averaging responses across all 6 items (α = 0.91 in 2006, range 1-5). After the 2006 wave, the HRS switched to a more expansive measure of positive affect based on the Positive and Negative Affect Schedule–Expanded Form (PANAS-X; Watson & Clark, 1994). It included the following 13 items: determined, enthusiastic, active, proud, interested, happy, attentive, content, inspired, hopeful, alert, calm, and excited. An overall score was derived by averaging responses across all 13 items (α = 0.92, range 1-5). A limitation of this study is that affect was measured in a different way during only the first wave of the study. However, scores were standardized and both the prior and current measures of affect operate very similarly (e.g., similar correlations with other variables, similar pattern of descriptive statistics).

*Life satisfaction.* Life satisfaction was assessed with the 5-item Satisfaction with Life Scale (Diener et al., 1985). The scale has shown excellent psychometric properties in prior work. Using a 7-point response scale from 1 (strongly disagree) to 7 (strongly agree), participants were asked the extent to which they agreed with statements such as, “In most ways my life is close to ideal.” Responses to all items were averaged to create a composite score, with higher scores indicating higher life satisfaction (α = 0.88, range 1-7).

*Optimism.* Optimism was assessed with the Life Orientation Test-Revised, which has good discriminant and convergent validity, as well as good reliability (Scheier et al., 1994). Using a 6-point response scale from 1 (strongly disagree) to 6 (strongly agree), participants were asked the degree to which they agreed with statements such as, “In uncertain times, I usually expect the best.” Negatively worded items were reverse coded and responses to all items were averaged to create an overall score, with higher scores indicating higher optimism (α = 0.75, range 1-6).

*Purpose in life.* Purpose in life was assessed with a 7-item purpose in life subscale from Ryff’s Psychological Well-Being Scale (Ryff & Keyes, 1995). The 7-item subscale has been validated in prior work and has shown good psychometric properties (Abbott et al., 2006). Using a 6-point response scale from 1 (strongly disagree) to 6 (strongly agree), participants were asked the degree to which they agreed with statements such as, “I have a sense of direction and purpose in my life.” Negatively worded items were reverse coded, and all items were averaged to create a composite score, with higher scores indicating higher purpose (α = 0.77, range 1-6).

*Personal mastery.* Mastery was assessed with 5 items derived from Lachman and Weaver (1998). The measure has good discriminant and convergent validity, and good reliability (Lachman & Weaver, 1998). Using a 6-point response scale from 1 (strongly disagree) to 6 (strongly agree), participants were asked the degree to which they agreed with statements such as, “I can do just about anything I really set my mind to.” All items were averaged to create a composite score, with higher scores indicating higher mastery (α = 0.90, range 1-6).

*Health mastery.* Participants were asked, “How would you rate the amount of control you have over your health these days?” on a 0 (no control at all) to 10 (very much control) scale.

*Financial mastery.* Participants were asked, “How would you rate the amount of control you have over your financial situation these days?” on a 0 (no control at all) to 10 (very much control) scale.

**Psychological Distress**

*Depressive symptoms and depression.* Depressive symptoms were measured using the Center for Epidemiologic Studies Depression Scale (CESD; Radloff, 1977). This scale has been validated in the HRS (Steffick, 2000). Participants indicated the presence of 8 depressive symptoms (e.g., “Much of the time during the past week, I felt depressed”) over the past week (yes/no). All items were summed, with higher scores indicating higher depressive symptoms (α = 0.80, range 0-8). Participants with scores of ≥ 4 were classified as having depression, as done previously (Steffick, 2000). No depression was the reference group. Prior work has suggested that the cutoff value of 4 would produce results similar to the 16-item cutoff when using the full (20-item) CESD scale (Steffick, 2000).

*Hopelessness.* Hopelessness was assessed with a 4-item questionnaire from two previously validated scales (Beck et al., 1974; Everson et al., 1997). Using a 6-point response scale from 1 (strongly disagree) to 6 (strongly agree), participants were asked the degree to which they agree with statements such as, “The future seems hopeless to me” and “I can’t believe that things are changing for the better.” All items were averaged to create a composite score (α = 0.86, range 1-6), with higher scores indicating more hopelessness.

*Negative affect*. Negative affect was measured (in 2006 only) with a 6-item scale originally developed for use in the Midlife in the United States Study (Mroczek & Kolarz, 1998; Watson et al., 1988). The scale assessed how often the participant felt “so depressed that nothing could cheer you up,” “hopeless,” “restless or fidgety,” “that everything was an effort,” “worthless,” and “nervous” over the past 30 days. Response categories ranged from 1 (all of the time) to 5 (none of the time). Responses were reverse scored, so that a higher score indicated higher negative affect. An overall score was derived by averaging responses across all 6 items (α = 0.87 in 2006, range 1-5). After the 2006 wave, the HRS switched to a more expansive measure of negative affect based on the PANAS-X (Watson & Clark, 1994). It included the following 12 items: afraid, upset, guilty, scared, frustrated, bored, hostile, jittery, ashamed, nervous, sad, and distressed. An overall score was derived by averaging responses across all 12 items (α = 0.89, range 1-5). A limitation of this study is that affect was measured in a different way during only the first wave of the study. However, scores were standardized and both the prior and current measures of affect operate very similarly (e.g., similar correlations with other variables, similar pattern of descriptive statistics).

*Perceived constraints.* Perceived constraints were assessed with 5 other items derived from Lachman and Weaver (1998), and this measure has good discriminant and convergent validity, as well as good reliability (Lachman & Weaver, 1998). Using a 6-point response scale from 1 (strongly disagree) to 6 (strongly agree), participants were asked the degree to which they agreed with statements such as, “What happens in my life is often beyond my control.” All items were averaged to create an overall score, with higher scores indicating a higher sense of constraints on personal control (α = 0.87, range 1-6).

*Anxiety symptoms.* Anxiety was assessed using 5 of the 21 items in the Beck Anxiety Inventory (Beck et al., 1988). This inventory has been shown to differentiate between symptoms of depression and anxiety and has been validated in older adults (Wetherell & Areán, 1997). Participants were asked, “How often did you feel that way during the past week.” 1) “I had fear of the worst happening,” 2) “I was nervous,” 3) “I felt my hands trembling,” 4) “I had a fear of dying,” and 5) “I felt faint,” and could respond with 1 of 4 categories: 1) Never, 2) Hardly ever, 3) Some of the time, 4) Most of the time. The five responses were averaged, with higher scores indicating greater anxiety symptoms (α = 0.81, range 1-4).

*Trait anger & state anger.* Trait anger (anger-in) and state anger (anger-out) are the two dimensions along which the Spielberger Anger Expression Scale measures anger (Forgays et al., 1998). These two dimensions have been shown to be separate factors that are modestly correlated through a principal factor analysis with Promax rotation (Lee & Bierman, 2018). Trait anger is the predisposition to respond with anger across a variety of situations. To measure this variable, participants were asked to respond to four statements such as, “When I am feeling angry or mad, I keep things in.” State anger is a temporary behavioral reaction of anger and was measured through seven statements including, “When I am feeling angry or mad, I strike out at whatever infuriates me.” Participants gave responses on a 4-point response scale for each item: 1) Almost never, 2) Sometimes, 3) Often and 4) Almost always. Responses were averaged for trait anger (α = 0.80) and state anger (α = 0.82) separately, with higher scores indicating higher trait anger and state anger (range 1-4).

*Cynical hostility.* Cynical hostility was measured using 5 items from the Cook-Medley Hostility Inventory (Cook & Medley, 1954). The items were as follows: 1) “Most people dislike putting themselves out to help other people,” 2) “Most people will use somewhat unfair means to gain profit or an advantage rather than lose it,” 3) “No one cares much what happens to you,” 4) “I think most people would lie in order to get ahead,” and 5) “I commonly wonder what hidden reasons another person may have for doing something nice for me.” The first statement was written as, “Most people inwardly dislike putting themselves out to help other people” in the 2006 and 2008 questionnaire before being changed from 2010 onwards. Participants responded on a 6-point response scale from 1 (strongly disagree) to 6 (strongly agree). The scores were averaged (α = 0.78, range 1-6), with higher scores indicating higher cynical hostility.

*Stressful life events.* Stressful life events were measured using 5 questions that have been used in other widely-used self-report measures of life stress (Turner et al., 1995). Items included questions such as, “Have you been unemployed and looking for work for longer than 3 months at some point in the past five years?” While the questionnaire in 2008 onwards asked an additional question of, “Have you been the victim of fraud in the past five years?”, this was not included for the purposes of the present study to maintain consistency (since it was not included in the 2006 questionnaire). Participants answered each question with a yes or no. Responses (0 = no, 1 = yes) were summed, with higher values indicating a higher number of stressful life events.

*Financial strain.* Respondents were asked, “How difficult is it for (you/your family) to meet monthly payments on (your/your family’s) bills?” and response options included: 1) Not at all difficult, 2) Not very difficult, 3) Somewhat difficult, 4) Very difficult or 5) Completely difficult. Higher scores indicated more financial strain.

*Daily discrimination & major discrimination.* Items measuring daily discrimination and major discrimination were based on prior widely used discrimination assessments (Essed, 1991; Feagin, 1991; Williams et al., 1997). Daily discrimination was measured using 5 items that capture the frequency of the following experiences in the day-to-day lives of participants: 1) being treated with less courtesy or respect, 2) receiving poorer service in restaurants or stores, 3) people acting as if you are not smart, 4) people acting as if they are afraid of you, and 5) being threatened or harassed. Participants answered with one of the following response categories: 1) Almost every day, 2) At least once a week, 3) A few times a month, 4) A few times a year, 5) Less than once a year and 6) Never. Items were reverse-coded and averaged (α = 0.80, range 1-6) such that higher scores indicated higher daily discrimination. The item, “You receive poorer service or treatment than other people from doctors or hospitals” (introduced in 2008) was excluded in the present study to maintain consistency as it was not present in the 2006 questionnaire. Major discrimination was measured using 6 items (yes/no) to capture major instances of lifetime discrimination: 1) being unfairly dismissed from a job, 2) not being hired for a job, 3) being unfairly denied a promotion, 4) being prevented from moving to a neighborhood because the realtor refused to sell/rent to you, 5) being unfairly denied a bank loan, and 6) being unfairly stopped by the police. Responses were summed with higher scores indicating more experiences of major discrimination. One item (“Have you ever been unfairly denied health care or treatment?” (introduced in 2008)) was excluded in the present study to maintain consistency as it was not included in the 2006 questionnaire.

**Social Factors**

*Living with a partner/spouse.* Participants were asked, “Do you have a husband, wife, or partner with whom you live?”, and answered yes/no.

*Frequency of contact with children, other family, friends.* The frequency of contact respondents had with members in their social network was evaluated through 3 items each for contacts who had 1) children, 2) other family, and 3) friends. Participants were asked, “On average, how often do you do each of the following?” 1) “Meet up (include both arranged and chance meetings),” 2) “Speak on the phone,” and 3) “Write or email.” Possible response categories were as follows: 1) Three or more times a week, 2) Once or twice a week, 3) Once or twice a month, 4) Every few months, 5) Once or twice a year or 6) Less than once a year or never. The responses were recoded into the following categories: 0 = Never-every few months, 1 = 1-2x/month, 2 = 1-2x/week and 3 = 3 or more times/week. If participants reported not having personal relationships within a particular category (e.g., they reported not having children), they were assigned a value of 0 to the frequency of contact items for that category of relationship.

*Loneliness.* Loneliness was assessed with three items from the previously validated UCLA Loneliness Scale (Russell, 1996). Participants were asked, “How much of the time do you feel”: 1) “you lack companionship,” 2) “left out,” and 3) “isolated from others,” with response categories ranging from 1 (often) to 3 (hardly ever or never). Responses were reverse scored and averaged, with higher scores indicating higher loneliness (α = 0.80, range 1-3).

*Closeness with spouse.* One’s closeness with their spouse, if they had one, was assessed using a single question, “How close is your relationship with your spouse or partner?” Response options included: 1) Very close, 2) Quite close, 3) Not very close and 4) Not at all close. Responses were reverse coded to range from 1 (not at all close) to 4 (very close). In our imputed analyses, participants without a spouse were treated as having missing data (and therefore, data on closeness with spouse were imputed for these participants). To avoid a large drop in sample size for complete-case analyses, responses to the item were recoded into the following categories to include those without a spouse: 0 = not at all close or not very close, 1 = quite close or very close, and 2 = not applicable (does not have a spouse).

*Number of close children, close other family, close friends.* The quantity of close social ties was measured through the following 3 items: 1) “How many of your children would you say you have a close relationship with?”, 2) “How many of these family members would you say you have a close relationship with?”, and 3) “How many of your friends would you say you have a close relationship with?”

*Social support from spouse, children, other family, friends & social strain from spouse, children, other family, friends.* The social support and social strain associated with each category of social ties were assessed using 3 and 4 items, respectively. These items were based on those used in previous studies on social support (Schuster et al., 1990; Turner et al., 1983). Items assessing social support were as follows: 1) “How much do they really understand the way you feel about things?”, 2) “How much can you rely on them if you have a serious problem?” and 3) “How much can you open up to them if you need to talk about your worries?” The 4 items assessing social strain were: 1) “How often do they make too many demands on you?”, 2) “How much do they criticize you?”, 3) “How much do they let you down when you are counting on them?” and 4) “How much do they get on your nerves?” Response options for all 7 questions included: 1) A lot, 2) Some, 3) A little, or 4) Not at all. Scores were reverse coded and then averaged to create separate indexes for social support and social strain. Higher values indicated more social support or more social strain. This was done for social support from spouse (α = 0.81), children (α = 0.82), other family (α = 0.82), and friends (α = 0.84), as well as for social strain from spouse (α = 0.78), children (α = 0.77), other family (α = 0.78), and friends (α = 0.75).

In our imputed analyses, participants without a spouse were treated as having missing data (and therefore, data on social support/strain from a spouse were imputed for these participants). For each of the other relationship categories (i.e., children, other family, friends), participants should not have answered items about social support/strain for each category of relationship that they indicated they did not have (e.g., they reported not having children) and therefore were treated as having missing data on those items (i.e., social support/strain for that category of relationship were imputed). In complete-case analyses, to avoid a large drop in sample size for spousal support/strain, responses were recoded into the following categories so that participants without a spouse were included: 0 = low support/strain from a spouse (average of ≤ 2.5 on spousal social support/strain items), 1 = high support/strain from a spouse (average of > 2.5 on the spousal social support/strain items), and 2 = not applicable (does not have a spouse). For each of the other relationship categories (i.e., children, other family, friends), participants without social support/strain data were treated as missing in complete-case analyses.

*Volunteering.* Respondents were asked, “Have you spent any time in the past 12 months doing volunteer work for religious, educational, health-related or other charitable organizations?” If they answered yes to this question, respondents were asked how many hours they volunteered. Responses were coded as: 0 = 0 hours, 1 = 1-49 hours, 2 = 50-99 hours, 3 = 100-199 hours, and 4 = ≥ 200 hours. Higher values indicated a greater amount of time spent volunteering.

*Helping friends, neighbors, relatives.* Respondents were asked, “Have you spent any time in the past 12 months helping friends, neighbors, or relatives who did not live with you and did not pay you for the help?” If they answered yes to this question, respondents were asked how many hours they spent helping. Responses were coded as: 0 = 0 hours, 1 = 1-49 hours, 2 = 50-99 hours, 3 = 100-199 hours, and 4 = ≥ 200 hours. Higher values indicated a greater amount of time spent helping others.

*Social status ladder & change in social status ladder.* The MacArthur scale of subjective social status was used to evaluate an individual’s own position on the social ladder (Adler et al., 2000). Participants were asked to think of a ladder on which the people at the top were best off and those at the bottom were worst off based on money, education level and job quality (e.g., having one of the best jobs vs. having the worst jobs or no job). The first item asked respondents to place themselves on the ladder (range 1-10). The second item asked, “Has your position on the ladder changed within the last two years?” Participants could answer 1) Yes, I have moved up, 2) Yes, I have moved down, or 3) No, my position has not changed. Responses were recoded into the following categories: 1 = downward movement, 2 = no change and 3 = upward movement.

**Work**

*In labor force.* Participants were asked, “Are you currently working?” An answer of 1 indicated “yes” while a 5 indicated “no”. Responses were recoded such that 1 = “In labor force” and 0 = “Not in labor force”.

## References

Abbott, R., Ploubidis, G., Huppert, F., Kuh, D., Wadsworth, M., & Croudace, T. (2006). Psychometric evaluation and predictive validity of Ryff’s psychological well-being items in a UK birth cohort sample of women. *Health and Quality of Life Outcomes*, *4*, 76. https://doi.org/10.1186/1477-7525-4-76

Adler, N. E., Epel, E. S., Castellazzo, G., & Ickovics, J. R. (2000). Relationship of subjective and objective social status with psychological and physiological functioning: Preliminary data in healthy white women. *Health Psychology*, *19*(6), 586–592. https://doi.org/10.1037//0278-6133.19.6.586

Beck, A. T., Epstein, N., Brown, G., & Steer, R. A. (1988). An inventory for measuring clinical anxiety: Psychometric properties. *Journal of Consulting and Clinical Psychology*, *56*(6), 893–897. https://doi.org/10.1037//0022-006x.56.6.893

Beck, A. T., Weissman, A., Lester, D., & Trexler, L. (1974). The measurement of pessimism: The Hopelessness Scale. *Journal of Consulting and Clinical Psychology*, *42*, 861–865. https://doi.org/10.1037/h0037562

Chou, R., Dana, T., Bougatsos, C., Fleming, C., & Beil, T. (2011). Screening adults aged 50 years or older for hearing loss: A review of the evidence for the U.S. preventive services task force. *Annals of Internal Medicine*, *154*(5), 347–355. https://doi.org/10.7326/0003-4819-154-5-201103010-00009

Cook, W. W., & Medley, D. M. (1954). Proposed hostility and Pharisaic-virtue scales for the MMPI. *Journal of Applied Psychology*, *38*(6), 414–418. https://doi.org/10.1037/h0060667

Crimmins, E. M., Kim, J. K., Langa, K. M., & Weir, D. R. (2011). Assessment of cognition using surveys and neuropsychological assessment: The Health and Retirement Study and the Aging, Demographics, and Memory Study. *The Journals of Gerontology: Series B*, *66B*(Suppl. 1), i162–i171. https://doi.org/10.1093/geronb/gbr048

Diener, E., Emmons, R. A., Larsen, R. J., & Griffin, S. (1985). The Satisfaction with Life Scale. *Journal of Personality Assessment*, *49*(1), 71–75. https://doi.org/10.1207/s15327752jpa4901_13

Essed, P. (1991). *Understanding everyday racism: An interdisciplinary theory*. SAGE Publications, Inc. https://doi.org/10.4135/9781483345239

Everson, S. A., Kaplan, G. A., Goldberg, D. E., Salonen, R., & Salonen, J. T. (1997). Hopelessness and 4-year progression of carotid atherosclerosis: The Kuopio Ischemic Heart Disease Risk Factor Study. *Arteriosclerosis, Thrombosis, and Vascular Biology*, *17*(8), 1490–1495. https://doi.org/10.1161/01.ATV.17.8.1490

Feagin, J. R. (1991). The continuing significance of race: Antiblack discrimination in public places. *American Sociological Review*, *56*(1), 101–116. https://doi.org/10.2307/2095676

Fisher, G. G., Faul, J. D., Weir, D. R., & Wallace, R. B. (2005). *Documentation of chronic disease measures in the Health and Retirement Study (HRS/AHEAD)*. Survey Research Center, University of Michigan. https://hrs.isr.umich.edu/sites/default/files/biblio/dr-009.pdf

Fisher, G. G., Hassan, H., Faul, J. D., Rodgers, W. L., & Weir, D. R. (2017). *Health and Retirement Study imputation of cognitive functioning measures: 1992 – 2014 (final release version)*. Survey Research Center, University of Michigan. https://hrs.isr.umich.edu/sites/default/files/biblio/COGIMPdd.pdf

Forgays, D. K., Spielberger, C. D., Ottaway, S. A., & Forgays, D. G. (1998). Factor structure of the State-Trait Anger Expression Inventory for middle-aged men and women. *Assessment*, *5*(2), 141–155. https://doi.org/10.1177/107319119800500205

Jenkins, C. D., Stanton, B.-A., Niemcryk, S. J., & Rose, R. M. (1988). A scale for the estimation of sleep problems in clinical research. *Journal of Clinical Epidemiology*, *41*(4), 313–321. https://doi.org/10.1016/0895-4356(88)90138-2

Katz, S., Ford, A. B., Moskowitz, R. W., Jackson, B. A., & Jaffe, M. W. (1963). Studies of illness in the aged: The Index of ADL: A standardized measure of biological and psychosocial function. *JAMA*, *185*(12), 914–919. https://doi.org/10.1001/jama.1963.03060120024016

Lachman, M. E., & Weaver, S. L. (1998). The sense of control as a moderator of social class differences in health and well-being. *Journal of Personality and Social Psychology*, *74*(3), 763–773. https://doi.org/10.1037/0022-3514.74.3.763

Langa, K. M., Plassman, B. L., Wallace, R. B., Herzog, A. R., Heeringa, S. G., Ofstedal, M. B., Burke, J. R., Fisher, G. G., Fultz, N. H., Hurd, M. D., Potter, G. G., Rodgers, W. L., Steffens, D. C., Weir, D. R., & Willis, R. J. (2005). The Aging, Demographics, and Memory Study: Study Design and Methods. *Neuroepidemiology*, *25*(4), 181–191. https://doi.org/10.1159/000087448

Lawton, M. P., & Brody, E. M. (1969). Assessment of older people: Self-maintaining and instrumental activities of daily living. *The Gerontologist*, *9*(3 Part 1), 179–186. https://doi.org/10.1093/geront/9.3_Part_1.179

Lee, Y., & Bierman, A. (2018). A longitudinal assessment of perceived discrimination and maladaptive expressions of anger among older adults: Does subjective social power buffer the association? *The Journals of Gerontology: Series B*, *73*(8), e120–e130. https://doi.org/10.1093/geronb/gbw110

Mroczek, D. K., & Kolarz, C. M. (1998). The effect of age on positive and negative affect: A developmental perspective on happiness. *Journal of Personality and Social Psychology*, *75*(5), 1333–1349. https://doi.org/10.1037/0022-3514.75.5.1333

Nagi, S. Z. (1976). An epidemiology of disability among adults in the United States. *The Milbank Memorial Fund Quarterly. Health and Society*, *54*(4), 439–467. https://doi.org/10.2307/3349677

Nandi, A., Glymour, M. M., & Subramanian, S. V. (2014). Association among socioeconomic status, health behaviors, and all-cause mortality in the United States. *Epidemiology*, *25*(2), 170–177. https://doi.org/10.1097/EDE.0000000000000038

National Institute on Alcohol Abuse and Alcoholism. (n.d.). *Drinking levels defined*. Retrieved July 7, 2021, from https://www.niaaa.nih.gov/alcohol-health/overview-alcohol-consumption/moderate-binge-drinking

Ofstedal, M. B., Fisher, G. G., & Herzog, A. R. (2005). *Documentation of cognitive functioning measures in the Health and Retirement Study*. Survey Research Center, University of Michigan. http://hrsonline.isr.umich.edu/sitedocs/userg/dr-006.pdf

Radloff, L. S. (1977). The CES-D Scale: A self-report depression scale for research in the general population. *Applied Psychological Measurement*, *1*(3), 385–401. https://doi.org/10.1177/014662167700100306

Rosow, I., & Breslau, N. (1966). A Guttman health scale for the aged. *Journal of Gerontology*, *21*(4), 556–559. https://doi.org/10.1093/geronj/21.4.556

Russell, D. W. (1996). UCLA Loneliness Scale (Version 3): Reliability, validity, and factor structure. *Journal of Personality Assessment*, *66*(1), 20–40. https://doi.org/10.1207/s15327752jpa6601_2

Ryff, C. D., & Keyes, C. L. M. (1995). The structure of psychological well-being revisited. *Journal of Personality and Social Psychology*, *69*(4), 719–727. https://doi.org/10.1037/0022-3514.69.4.719

Scheier, M. F., Carver, C. S., & Bridges, M. W. (1994). Distinguishing optimism from neuroticism (and trait anxiety, self-mastery, and self-esteem): A reevaluation of the Life Orientation Test. *Journal of Personality and Social Psychology*, *67*(6), 1063–1078. https://doi.org/10.1037/0022-3514.67.6.1063

Schuster, T. L., Kessler, R. C., & Aseltine, R. H. (1990). Supportive interactions, negative interactions, and depressed mood. *American Journal of Community Psychology*, *18*(3), 423–438. https://doi.org/10.1007/BF00938116

Steffick, D. E. (2000). *Documentation of affective functioning measures in the Health and Retirement Study*. Survey Research Center, University of Michigan. https://hrs.isr.umich.edu/sites/default/files/biblio/dr-005.pdf

Turner, R. J., Frankel, B. G., & Levin, D. M. (1983). Social support: Conceptualization, measurement, and implications for mental health. *Research in Community & Mental Health*, *3*, 67–111.

Turner, R. J., Wheaton, B., & Lloyd, D. A. (1995). The epidemiology of social stress. *American Sociological Review*, *60*(1), 104–125. https://doi.org/10.2307/2096348

Watson, D., & Clark, L. A. (1994). *The PANAS-X: Manual for the Positive and Negative Affect Schedule - Expanded Form*. https://doi.org/10.17077/48vt-m4t2

Watson, D., Clark, L. A., & Tellegen, A. (1988). Development and validation of brief measures of positive and negative affect: The PANAS scales. *Journal of Personality and Social Psychology*, *54*(6), 1063–1070. https://doi.org/10.1037/0022-3514.54.6.1063

Wetherell, J. L., & Areán, P. A. (1997). Psychometric evaluation of the Beck Anxiety Inventory with older medical patients. *Psychological Assessment*, *9*(2), 136–144. https://doi.org/10.1037/1040-3590.9.2.136

Williams, D. R., Yu, Y., Jackson, J. S., & Anderson, N. B. (1997). Racial differences in physical and mental Health: Socio-economic status, stress and discrimination. *Journal of Health Psychology*, *2*(3), 335–351. https://doi.org/10.1177/135910539700200305

World Health Organization. (1995). *Physical status: The use and interpretation of anthropometry*. https://apps.who.int/iris/bitstream/handle/10665/37003/WHO_TRS_854.pdf?sequence=1&isAllowed=y
