# Supplementary material for: Identifying pathways to religious service attendance among older adults: A lagged exposure-wide analysis
Source: PLoS One. 2022 Nov 29;17(11):e0278178. doi: 10.1371/journal.pone.0278178 (PMC9707744; doi:10.1371/journal.pone.0278178)
Supplement: S2 Appendix — (DOCX) [file pone.0278178.s002.docx]

Identifying Pathways to Religious Service Attendance: A Lagged Exposure-Wide Analysis in a Sample of Older U.S. Adults

**S2 APPENDIX**

**Proof Illustrating How Adjusting for the Prior Value of a Predictor Can Help Us Evaluate How “Changes” in the Predictor are Associated with Subsequent Religious Service Attendance**

Let Y be the religious service attendance outcome in 2014/2016, A_1_ the predictor under consideration in 2010/2012, A_0_ the prior value of the predictor in 2006/2008, and C the set of all other covariates in 2006/2008.

For a binary outcome, the regression model is: log{P[Y=1|a_0_, a_1_, c]} = v + b_0_a_0_ + b_1_a_1_+ b_2_’c, which, if the outcome is rare can be approximated by a logistic regression model, logit{P[Y=1|a_0_, a_1_, c]} = v + b_0_a_0_ + b_1_a_1_+ b_2_’c, or, if the outcome is common, by a modified Poisson model. Let Y_a_ denote the potential outcome Y for an individual under an intervention to set A_1_ to a. For an individual with baseline predictor A_0_=a_0_ and covariates c in 2006/2008, under the no-confounding (and positivity and consistency) and modeling assumptions, a change in predictor of d points A_0_=a_0_ to A_1_=a_0_+d in 2010/2012, rather than maintaining predictor of A_1_=a_0_ in 2010/2012, will give rise to an effect on the risk ratio scale of:

P[Y_a0+d_=1| A_0_=a_0_, c] / P[Y_a0_=1| A_0_=a_0_, c]

= P[Y_a0+d_=1| A_1_=a_0_+d, A_0_=a_0_, c] / P[Y_a0_=1| A_1_=a_0_, A_0_=a_0_, c]

= P[Y=1| A_1_=a_0_+d, A_0_=a_0_, c] / P[Y=1| A_1_=a_0_, A_0_=a_0_, c]

= exp[v + b_0_a_0_ + b_1_(a_0_+d) + b_2_’c] / exp[v + b_0_a_0_ + b_1_a_0_ + b_2_’c]

= exp(b_1_d)

where the first equality follows by the no-confounding assumption, the second by consistency, and the third by the statistical model.
