# Supplementary material for: Identifying pathways to religious service attendance among older adults: A lagged exposure-wide analysis
Source: PLoS One. 2022 Nov 29;17(11):e0278178. doi: 10.1371/journal.pone.0278178 (PMC9707744; doi:10.1371/journal.pone.0278178)
Supplement: S1 Table — (DOCX) [file pone.0278178.s003.docx]

Identifying Pathways to Religious Service Attendance: A Lagged Exposure-Wide Analysis in a Sample of Older U.S. Adults

**S1 TABLE**

|  | T_2_ | | | |
| --- | --- | --- | --- | --- |
|  | Rarely or never | 2-3x/month | 1x/week | > 1x/week |
| T_0_ |  |  |  |  |
| Rarely or never | 84.1 | 6.7 | 7.2 | 2.0 |
| 2-3x/month | 43.3 | 26.1 | 23.7 | 7.0 |
| 1x/week | 17.8 | 12.9 | 54.6 | 14.7 |
| > 1x/week | 9.6 | 8.1 | 35.0 | 47.3 |

S1 Table. Changes in Religious Service Attendance from T_0_ to T_2_^a,b^

^a^The percentage of people in category 1, 2, 3, or 4 at T_0_ who end up in category 1, 2, 3, or 4 at T_2_.

^b^Cumulative percentage in the second row (2-3x/month) does not add up to 100% due to rounding.
