# Supplementary material for: Identifying pathways to religious service attendance among older adults: A lagged exposure-wide analysis
Source: PLoS One. 2022 Nov 29;17(11):e0278178. doi: 10.1371/journal.pone.0278178 (PMC9707744; doi:10.1371/journal.pone.0278178)
Supplement: S7 Table — (DOCX) [file pone.0278178.s009.docx]

Identifying Pathways to Religious Service Attendance: A Lagged Exposure-Wide Analysis in a Sample of Older U.S. Adults

**S7 TABLE**

S7 Table. Complete-Case Analyses for Candidate Predictors (T_1_) of Subsequent Religious Service Attendance (T_2_)^a,b,c^

| Candidate predictor | Risk ratio | 95% confidence interval |
| --- | --- | --- |
| **Health behaviors** |  |  |
| Frequent physical activity | 1.01 | 0.92, 1.12 |
| Smoking | 0.79 | 0.58, 1.08 |
| Heavy drinking | 1.02 | 0.78, 1.33 |
| Sleep problems | 1.02 | 0.91, 1.15 |
| **Physical health** |  |  |
| Diabetes | 0.98 | 0.82, 1.17 |
| Hypertension | 0.99 | 0.85, 1.16 |
| Stroke | 1.12 | 0.89, 1.41 |
| Cancer | 1.09 | 0.90, 1.33 |
| Heart disease | 1.00 | 0.85, 1.19 |
| Lung disease | 0.99 | 0.75, 1.30 |
| Arthritis | 0.97 | 0.81, 1.15 |
| Overweight/obese | 1.05 | 0.91, 1.20 |
| Physical functioning limitations | 0.88 | 0.77, 1.00 |
| Cognitive impairment | 0.95 | 0.84, 1.09 |
| Chronic pain | 0.96 | 0.87, 1.06 |
| Self-rated health | 1.05 | 0.99, 1.12 |
| Hearing | 1.01 | 0.95, 1.07 |
| Eyesight | 1.01 | 0.96, 1.06 |
| **Psychological well-being** |  |  |
| Positive affect | 1.03 | 0.97, 1.09 |
| Life satisfaction | 1.03 | 0.97, 1.09 |
| Optimism | 1.02 | 0.96, 1.08 |
| Purpose in life | 1.03 | 0.97, 1.09 |
| Personal mastery | 0.99 | 0.94, 1.05 |
| Health mastery | 1.04 | 0.98, 1.10 |
| Financial mastery | 1.02 | 0.97, 1.08 |
| **Psychological distress** |  |  |
| Depression | 0.97 | 0.82, 1.14 |
| Depressive symptoms | 1.01 | 0.95, 1.07 |
| Hopelessness | 0.95 | 0.89, 1.01 |
| Negative affect | 1.01 | 0.95, 1.07 |
| Perceived constraints | 0.97 | 0.92, 1.03 |
| Anxiety symptoms | 0.98 | 0.93, 1.05 |
| Trait anger | 0.97 | 0.92, 1.02 |
| State anger | 1.01 | 0.95, 1.07 |
| Cynical hostility | 0.98 | 0.92, 1.03 |
| Stressful life events | 1.00 | 0.95, 1.05 |
| Financial strain | 0.98 | 0.92, 1.04 |
| Daily discrimination | 1.01 | 0.95, 1.07 |
| Major discrimination | 1.00 | 0.94, 1.06 |
| **Social factors** |  |  |
| Living with spouse/partner | 0.96 | 0.82, 1.14 |
| Contact with children |  |  |
| < Every few months | Reference | Reference |
| 1-2x/month | 1.02 | 0.80, 1.29 |
| 1-2x/week | 0.97 | 0.78, 1.21 |
| ≥ 3x/week | 0.95 | 0.76, 1.20 |
| Contact with other family |  |  |
| < Every few months | Reference | Reference |
| 1-2x/month | 0.96 | 0.85, 1.10 |
| 1-2x/week | 0.98 | 0.86, 1.11 |
| ≥ 3x/week | 0.96 | 0.83, 1.11 |
| Contact with friends |  |  |
| < Every few months | Reference | Reference |
| 1-2x/month | 1.07 | 0.90, 1.27 |
| 1-2x/week | 1.13 | 0.96, 1.32 |
| ≥ 3x/week | 1.11 | 0.94, 1.31 |
| Loneliness | 1.03 | 0.97, 1.10 |
| Closeness with spouse | 1.05 | 0.98, 1.12 |
| Number of close children | 1.01 | 0.97, 1.05 |
| Number of close other family | 1.00 | 0.96, 1.04 |
| Number of close friends | 1.02 | 0.98, 1.06 |
| Social support from spouse | 1.02 | 0.95, 1.09 |
| Social support from children | 1.00 | 0.94, 1.06 |
| Social support from other family | 1.01 | 0.95, 1.06 |
| Social support from friends | 1.04 | 0.98, 1.10 |
| Social strain from spouse | 1.01 | 0.95, 1.07 |
| Social strain from children | 1.01 | 0.95, 1.07 |
| Social strain from other family | 1.00 | 0.95, 1.06 |
| Social strain from friends | 1.03 | 0.98, 1.09 |
| Volunteering |  |  |
| 0 hours | Reference | Reference |
| 1-49 hours | 1.32 | 1.17, 1.50*** |
| 50-99 hours | 1.39 | 1.21, 1.59*** |
| 100-199 hours | 1.34 | 1.16, 1.55*** |
| ≥ 200 hours | 1.35 | 1.14, 1.59*** |
| Helping friends/neighbors/relatives |  |  |
| 0 hours | Reference | Reference |
| 1-49 hours | 1.03 | 0.93, 1.14 |
| 50-99 hours | 1.03 | 0.91, 1.18 |
| 100-199 hours | 1.03 | 0.88, 1.20 |
| ≥ 200 hours | 1.03 | 0.86, 1.24 |
| Social status ladder | 1.02 | 0.96, 1.08 |
| Change in social status ladder |  |  |
| Moved down | Reference | Reference |
| No change | 0.98 | 0.85, 1.13 |
| Moved up | 1.05 | 0.86, 1.27 |
| **Work** |  |  |
| In labor force | 1.04 | 0.92, 1.17 |

*Note*. *N* for analyses ranged from = 3,022 to 5,457. **p* < .05 before Bonferroni correction; ** *p* < .01 before Bonferroni correction; ****p* < .05 after Bonferroni correction (the *p*-value cut-off for Bonferroni correction is *p* = .05/60 predictors: *p* < .00083333).

^a^The analytic sample was restricted to those who had participated in 2006/2008 (T_0_). Candidate predictors were assessed in 2010/2012 (T_1_), and the outcome (religious service attendance) was assessed in 2014/2016 (T_2_). All models controlled for the full set of covariates, including sociodemographic factors, personality traits, prior values of all candidate predictors (except for the depression variable, which was constructed from the depressive symptoms variable), and prior religious service attendance, each of which was assessed at T_0_ (see Table 1).

^b^All continuous candidate predictors were standardized (mean = 0, standard deviation = 1).

^c^An exposure-wide analytic approach was used, and a separate model was run for each candidate predictor. Because religious service attendance was a binary outcome with a prevalence of ≥ 10%, we ran a generalized linear model with a log link and Poisson distribution to estimate a risk ratio.
